# Disruption of DNA methylation via *S*-adenosylhomocysteine is a key process in high incidence liver carcinogenesis

**DOI:** 10.1186/1868-7083-5-S1-S9

**Published:** 2013-08-19

**Authors:** Leda Mirbahai, Tim D Williams, Guangliang Yin, Andrew D Southam, Ulf Sommer, Ning Li, John P Bignell, Brett P Lyons, Mark R Viant, James K Chipman

**Affiliations:** 1 School of Biosciences, University of Birmingham, Edgbaston, Birmingham, UK; 2 Beijing Institute of Genomics, Chinese Academy of Sciences, Beijing 100029, China; 3 NERC Biomolecular Analysis Facility – Metabolomics Node (NBAF-B), School of Biosciences, University of Birmingham, Edgbaston, Birmingham, UK; 4 Cefas, Weymouth Laboratory, Weymouth, Dorset, UK

## 

Modifications in histologically normal tissue distal to tumours are increasingly evident and the role of such molecular events in tumour susceptibility or in response to presence of a tumour is unclear. We have exploited the ability to explain distal tissue modifications in the dab fish (*Limanda limanda*) which has an unprecedented high occurrence of hepatic adenoma (up to 20%) when analysed from the natural environment. To investigate this, three tissue categories of hepatocellular adenoma, histologically normal liver tissue distal to tumours and livers of non-tumour-bearing dab were used. A multi-“omics” approach was used to provide a comprehensive understanding of the key molecular abnormalities. A remarkable and consistent global hypomethylation, modification of CpG island methylation, gene expression and disruption of one-carbon metabolism was discovered in normal tissue distal to tumours compared to livers of non-tumour-bearing fish. The mechanism of this disruption is linked, not to depletion of *S*-adenosylmethionine, as is often a feature of mammalian tumours, but to a decrease in choline and elevated *S*-adenosylhomocysteine, a potent inhibitor of DNA methytransferase. This novel feature of normal-appearing tissue of tumour-bearing fish helps to understand the unprecedentedly high incidence of tumours in fish sampled from the field and adds weight to the controversial epigenetic progenitor model of tumourigenesis.

